# Bioinformatics reveal macrophages marker genes signature in breast cancer to predict prognosis

**DOI:** 10.1080/07853890.2021.1914343

**Published:** 2021-06-30

**Authors:** Ying Li, Xin Zhao, Qiang Liu, Yujie Liu

**Affiliations:** a Guangdong Provincial Key Laboratory of Malignant Tumor Epigenetics and Gene Regulation, Sun Yat-sen Memorial Hospital, SunYat-sen University, Guangzhou, China; b Breast Tumor Center, Sun Yat-sen Memorial Hospital, Sun Yat-sen University, Guangzhou, China; c Department of Breast Surgery, Affiliated Cancer Hospital of Zhengzhou University, Henan Cancer Hospital, Zhengzhou, China

**Keywords:** Breast cancer, macrophages marker genes, prognostic signature, bioinformatics

## Abstract

**Background:**

Breast cancer is a pivotal cause of global women cancer death. Immunotherapy has become a promising means to cure breast cancer. As constitutes of immune microenvironment of breast cancer, macrophages exert complicated functions in the tumour development and treatment. This study aims to develop a prognostic macrophage marker genes signature (MMGS).

**Methods:**

Single cell RNA sequence data analysis was performed to identify macrophage marker genes in breast cancer. TCGA database was used to construct MMGS model as a training cohort, and GSE96058 dataset was used to validate the MMGS as a validation cohort.

**Results:**

Genes included in the MMGS model were: SERPINA1, CD74, STX11, ADAM9, CD24, NFKBIA, PGK1. MMGS risk score stratified by overall survival of patients divided them into high- and low-risk groups. And MMGS risk score remained independent prognostic factor in multivariate analysis after adjusting for classical clinical factors in both training and validation cohorts. Besides, hormone receptors negative and human epidermal growth factor receptor 2 (HER2) positive patients had higher risk score. MMGS showed better distinguishing capability between high-risk and low-risk groups in hormone receptor positive and HER2 negative subgroup.

**Conclusion:**

MMGS provides a new understanding of immune cell marker genes in breast cancer prognosis and may offer reference for immunotherapy decision for breast cancer patients.

## Introduction

Breast tumour is one of the pivotal cause of female cancer death [1]. Although the survival outcomes have significantly improved over the past decades, patients with metastatic breast cancer still show poor prognosis. Accumulating evidence indicates that interplay between tumour cells and stromal cells, including diversity of immune cells, fibroblasts, etc., as well as the tumour microenvironment (TME) evolve during the progression of disease exert a crucial impact on patients’ survival and response to therapies [2]. The extensive TME heterogeneity contributes to the difficulty of cancer management. In recent years, the achievements of overall survival prolongation in breast cancer patients through immunotherapy opens the possibility for new treatment options. However, several clinical trials indicate responses to immune checkpoint inhibitor monotherapy or other combination therapies vary in breast cancer subtypes. Immunological parameters, including tumour-infiltrating lymphocytes (TILs) and stromal cell phenotypes, may be of great significance in breast cancer treatment [3–5].

As the most abundant immune-related stromal cells in the tumour microenvironment, tumour-associated macrophages (TAMs) can be phenotypically polarized in response to different microenvironmental signals to mount specific functional programs. Macrophages play an indispensable role in fighting off diseaseas part of the immune system and marshalling other immune cells to the scene. In cancer, macrophage phagocytosis leads to tumour elimination, inflammasome activation and antigen presenting which may induce adaptive immunity against tumors [6,7]. Next to their antineoplastic effects, macrophages also contribute to tumour progression, metastasis and resistance to therapy [6,8–10]. Our previous study showed that macrophages after antibody-dependent cellular phagocytosis (ADCP) increase the expression of PD-L1 and IDO, two immune checkpoint molecules, and phenotypically transit to immunosuppressive state [11]. Also, TAM can induce chemotaxis of circulating naïve CD4^+^ T cells to breast cancer that differentiate into Tregs *in situ* and lead to immunosuppression [12]. In addition, in tumour tissue the TAM-like phenotype of macrophages activated by mesenchymal-type breast cancer cells will, in turn, produce CCL18 to induce EMT of cancer cells, forming a positive feedback loop and promoting breast cancer metastasis [13,14]. Taken together, these studies suggest the functional plasticity of macrophages reflects the efficacy of therapy and prognosis of breast cancer. Therefore, it is necessary to better understand the gene expression profiles of immune cells, especially macrophages, in breast cancer and their associations with prognosis and therapeutic prediction.

In the past 15 years, different gene assays have been developed and validated in multiple clinical trial to stratify patients into different risk groups by analysing the abundance of diverse target gene combinations. These multi-gene expression assays like Oncotype DX [15], MammaPrint [16] and PAM50 Prosigna Assay [17] now have been widely used to predict the recurrence risk of breast cancer patients. Application of these gene-expression signatures has helped physician’ clinical practices. However, genes detected in these assays were mainly proliferation-related genes and restrict to tumour cells. None of the current widely used multi-gene expression assays include prognosis-related immune cell marker genes, which may be crucial in the immunotherapy and other combination therapies.

The application of single-cell RNA-sequencing (scRNA-seq) offers researchers an effective tool to analyse and understand the mechanisms of oncogenesis and heterogeneity of breast cancer to pave the way for individualized management [18,19]. As has been reported before, scRNA-seq analysis of immune cells in the breast cancer microenvironment helped to discover some specific immune cell subpopulations which serve as potential targets of immunotherapy [20,21]. Selecting immune cell marker genes for molecular signatures might be an effective approach to predict long-term prognosis and therapeutic benefit of breast cancer patients. In this study, we exploited scRNA-seq profiles from the Gene Expression Omnibus (GEO) and the Cancer Genome Atlas (TCGA) database to construct macrophage marker genes signature (MMGS) for breast cancer. Then, we validated the prognostic value of MMGS in the GSE96058 database.

It has been reported that gene transcription could be regulated by DNA methylation by recruiting repression proteins or by inhibiting transcription factors binding to DNA [22,23], we further analysed the correlation between mRNA expression and DNA methylation of MMGS.

## Method

### Data source and acquisition

ScRNA-seqdata in the form of RSEM normalized counts from six primary triple-negative breast cancers was downloaded fromGSE118389 dataset (https://www.ncbi.nlm.nih.gov/geo/query/acc.cgi?acc=GSE118389) [18], and used to screen macrophages marker genes. Normalized RNA sequencing data in the form of log_2_ (FPKM + 1) was downloaded from UCSC Xena (https://xenabrowser.net/datapages/) for further survival-related genes screening and model construction. Individual patient files and mRNA expression raw data were obtained from TCGA data portal (https://portal.gdc.cancer.gov/). To validate the prognostic ability of the constructed model, normalized RNA sequencing data in the form of log_2_ (FPKM + 0.1) was downloaded from GSE96058 dataset (https://www.ncbi.nlm.nih.gov/geo/query/acc.cgi?acc=GSE96058) [24,25]. DNA methylation raw data were obtained from TCGA data portal.

### Identification of macrophages marker genes and functional analysis

ScRNA-seq data analysis was performed by using “Seurat”, “SingleR” packages [26]. Cells with more than 5% of mitochondrial gene were removed [27]. Cells with number of gene mapped less than 200 and clusters with cell counts less than 5 were moved. We performed principal component analysis (PCA) using the most 1500 variable genes in the dataset in order to visualize transcriptional variability over the complete scRNA-seq dataset. T-distributed Stochastic Neighbour Embedding (t-SNE) was used for further dimensional reduction of the significant principal components [27]. Genes that exhibited a |log_2_ (fold change)|>0.8 and adjusted *p* value < .01 were considered as the marker genes. Kyoko Encyclopaedia of Genes and Genomes (KEGG) pathway enrichments and gene ontology (GO) analysis were conducted by using “ClusterProfiler” [28], “org.Hs.eg.db”, “GOplot” [29], “enrichplot” packages.

### Construction and validation of macrophages marker genes prognosis risk model

Cases with follow-up time more than 30 days in the TCGA database were included to build the risk score model. Macrophages marker genes expression data downloaded from UCSC Xena were merged with overall survival (OS) time and status for each case. To screen out the most significant genes account for OS, *p* value was set as less than .01 in the univariate Cox analysis. Statistically significant genes in the univariate Cox analysis were included to build multivariate Cox proportional hazards model. Further, we detected whether the genes in the signature model differentially expressed between the tumour tissue and tumour adjacent normal tissue in the TCGA dataset. X-tile software (version 3.6.1) was used to define the optimum cut-off value for risk scores based on the association with OS [30].”Survival”, “survminer” packages were used to construct Kaplan–Meier survival curves to evaluate survival differences between high-risk and low-risk groups both in the training and validation set. Then the capacity of the risk score to predict OS was tested by adjusting for classical clinical variables in the multivariate Cox model in both training and validation groups. Furthermore, time-dependent receiver operating characteristic (ROC) curve, area under the curve (AUC) and the concordance index (C-index) were performed to evaluate the discrimination power of this model [31]. Then, the heatmaps of these model genes in both training and validation groups were performed.

### Correlation between gene methylation and expression

Perl 64 was used to merge gene methylation and gene expression data. Then we analysed the correlation between methylation and gene expression by using correlation test in R [32].

### Statistical analysis

GSE96058 dataset normalized RNA sequencing data were transformed into the same format as TCGA data before model validation. All figure construction in this study was conducted by using R package software (version 4.0.3). Univariate and multivariate Cox regression model were performed by using “survival”, “survminer” packages. Wilcox test was used to determine statistical differences of categorical variables. Genes included in the multivariate analysis were selected through stepwise Cox regression analysis with both directions. Bootstrap method was used to perform internal validation in the TCGA dataset. The survival curves were measured by the Kaplan–Meier method and the significance of disparity was assessed by log-rank test. “timeROC”, “boot” packages were used to evaluate the prediction capacity of the prognostic model. Pearson correlation coefficient |r|>0.3 and *p* < .05 were defined as closely correlated.

## Results

### Identification of macrophages marker genes expression profiles and function annotation

**Figure 1.**
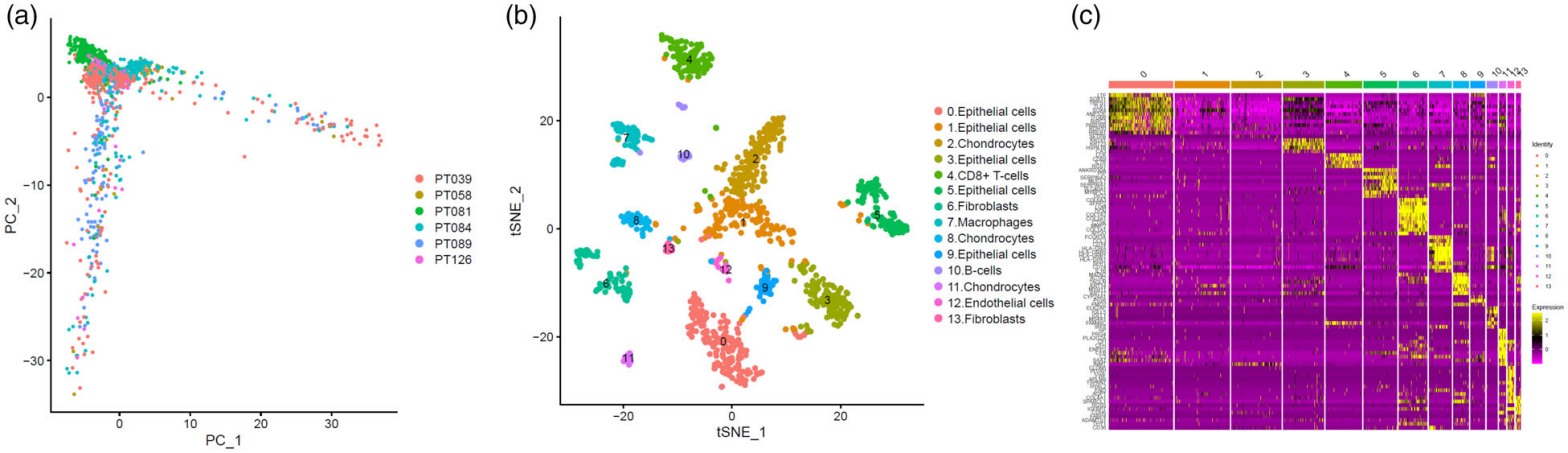
Identification macrophages marker genes by single cell sequence analysis. (a) PCA plot coloured by various samples. (b) t-SNE plot coloured by various cell types. (c) identification marker genes of different cell types.

According to the screening criteria, a total of 1205 cells from 6 samples were analysed to identify and characterize cell populations. We first reduced the dimensionality of the data by PCA by using the 1500 variable genes (Figure 1(a)). We then assigned cell-type identities by cross-referencing differentially expressed genes in each cluster with previously reported cell-type-specific marker genes (with |log_2_ (fold change)|>0.8 as well as adjusted *p* value <.01) [18,33], and identified 14 cell clusters using Seurat. Cells in cluster 7 expressing macrophage-specific markers were classified as macrophages (Figure 1(b)). We also found that this cluster had distinct gene expression profiles with a subset of genes differentially expressed between the 14 clusters (Figure 1(c)). As a result, we found out breast cancer-related 314 macrophage marker genes.

**Figure 2.**
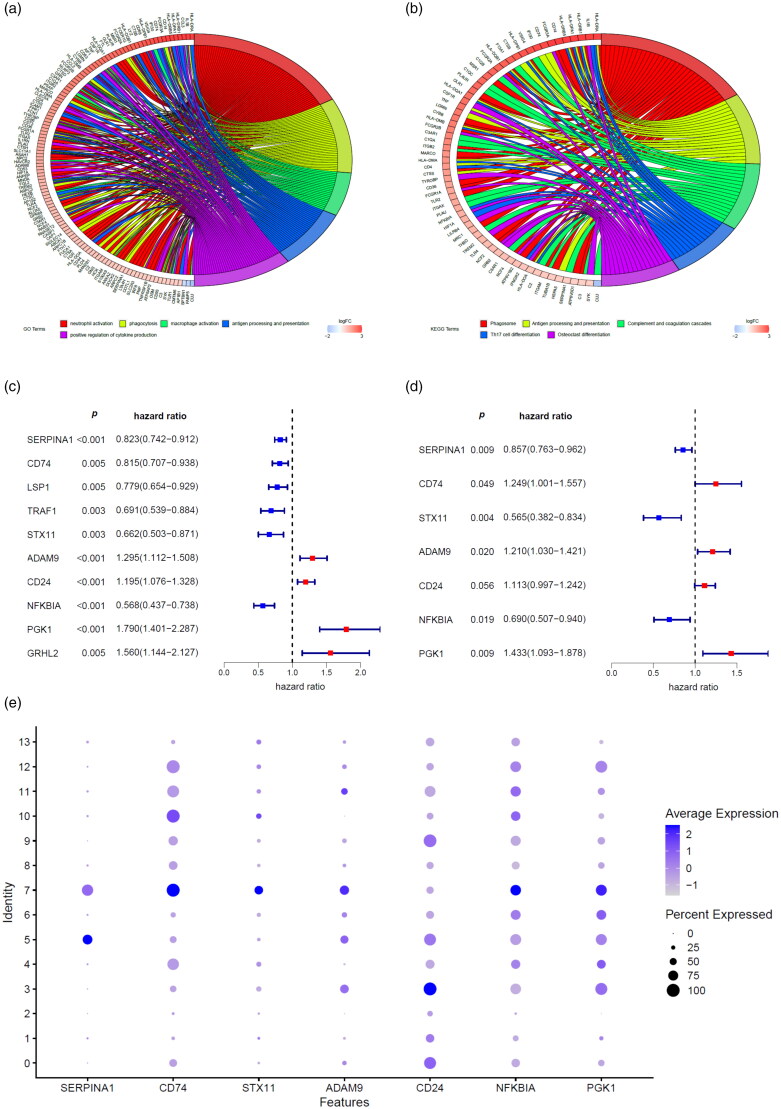
Screen of macrophage marker genes signature. GO analysis (a) and KEGG pathway enrichment (b) of macrophages marker genes were performed to explore the biological function of these marker genes. Macrophages marker genes related risk score model was developed by univariate analysis (c) and multivariate analysis (d) of macrophages marker genes that were associated with overall survival of breast cancer patients in the TCGA database. (e) Dot plot showing the expression of macrophages marker genes included in the risk score model in various cell types. Dot intensity of colour indicates the average expression in a particular cluster and dot size represents the percent of cells expressing the gene in that cluster. Cluster 7 represents macrophages.

A total of 314 macrophage marker genes were screened from GSE118389 dataset according to the screening criteria. Then GO analysis (Figure 2(a)) and KEGG pathway enrichment (Figure 2(b)) were performed to explore the biological function of these marker genes. We found that macrophages marker genes were mostly involved in the process of neutrophil activation, phagocytosis, macrophage activation, etc. (Figure 2(a)). The KEGG pathway enrichment demonstrated that macrophages marker genes primarily participated in the pathway of phagosome, antigen processing and presentation, complement and coagulation cascades, etc.

### Construction of prognostic macrophage marker genes signature (MMGS)

In order to identify a macrophages gene prognostic signature, we used TCGA cohort as the training set. According to the screen criteria previously described, there were a total of 1034 patients included in the training cohort with follow-up time ranging from 31 to 8605 days, with a median follow-up of 865 days. The clinicopathological characteristics of included cases were shown in Supplemental Table S1.

The univariate Cox proportional hazards regression analysis of TCGA database revealed that10macrophage marker genes with significantly associated with OS were selected as candidate genes of MMGS (Figure 2(c)). These 10 marker genes were then incorporated into a multivariate Cox proportional hazards regression model to determine the genes and their coefficients. Ultimately, 7 macrophage marker genes were included in the MMGS model (Figure 2(d)). The risk score which was used to predict prognosis was given as follows: MMGS risk score = SERPINA1expression × (−0.155) + CD74expression × 0.222 + STX11expression × (−0.572) + ADAM9expression × 0.190 + CD24expression × 0.107 + NFKBIAexpression × (−0.371) + PGK1expression × 0.360. The relative expression of the 7 marker genes in various clusters were shown in Figure 2(e), and the marker genes except CD24 were relatively higher in the macrophages cluster (cluster 7) compared to the other clusters as a whole.

Since macrophages in TME were activated to pro-tumoral phenotype, we assumed the MMGS risk score would discriminate macrophage characters between tumour and normal tissues. We detected the relative marker genes expression between tumour and tumour adjacent normal tissue. As shown in Supplemental Figure 1, ADAM9 and SERPINA1 expression between tumour and tumour adjacent normal tissue were not significantly different (*p* = .634, .491, respectively). CD24, CD74, PGK1 showed significantly higher expression in tumour tissue compared with tumour adjacent normal tissue, while STX11 and NFKBIA showed the opposite trend (*p* < .001 for all). Risk score showed significantly higher expression in tumour tissue compared with tumour adjacent normal tissue (*p* < .001).

**Figure 3.**
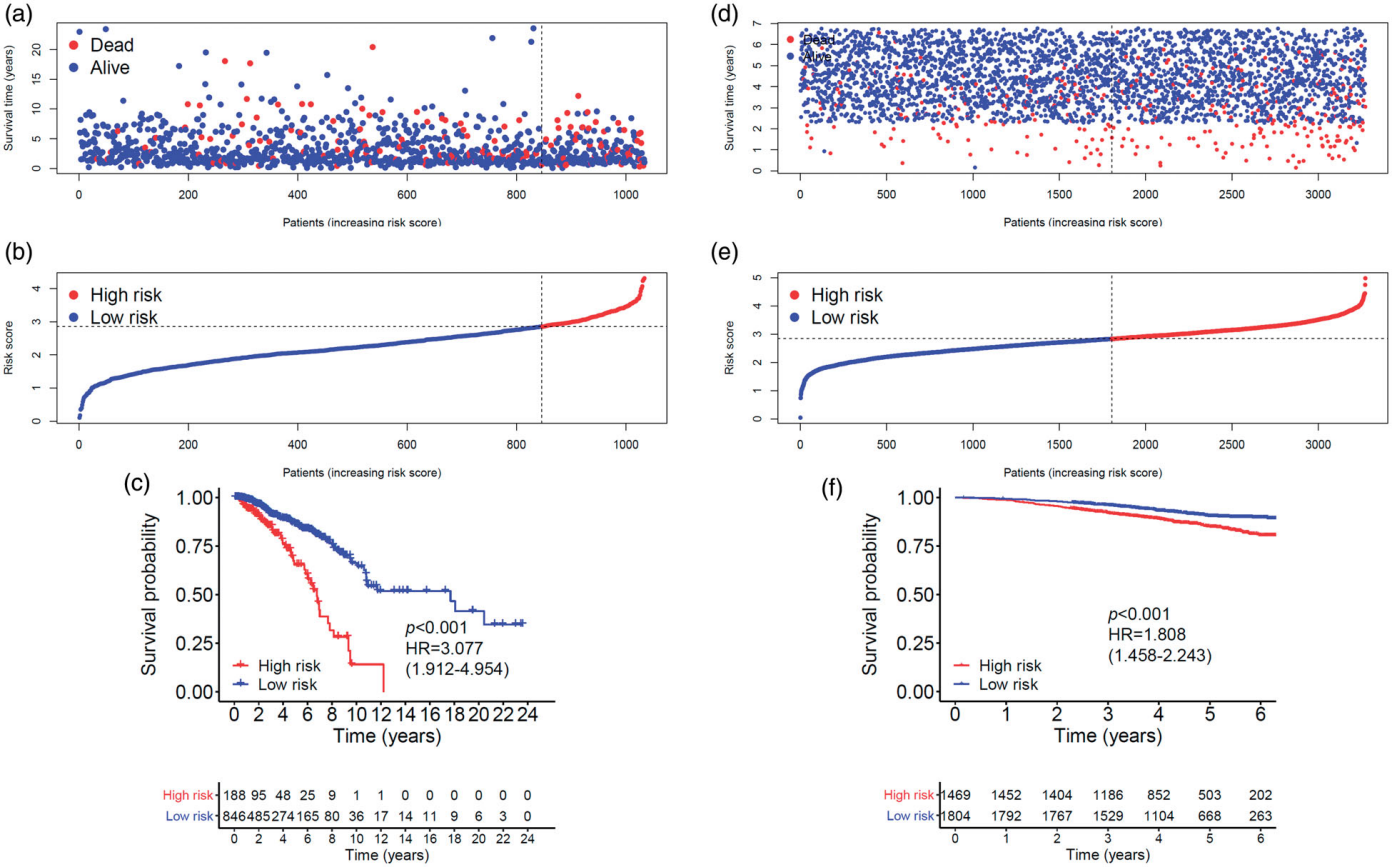
Breast cancer patients’ survival status, risk score distribution and Kaplan–Meier curves of OS in the TCGA database and the GSE96058 validation dataset. (a) Breast cancer patients were separated into high-risk and low-risk groups with the cut-off of risk score generated by X-tile software. (b) Breast cancer patients survival status and risk score distribution in the TCGA database. (c) Kaplan–Meier curves of OS between high-risk and low-risk groups in the TCGA database. (d) Breast cancer patients in the GSE96058 validation set were separated into high-risk and low-risk groups with the same cut-off in the TCGA database. (e) Breast cancer patients survival status and risk score distribution in the GSE96058 validation set. (f) Kaplan–Meier curves of OS between high-risk and low-risk groups in the GSE96058 validation set.

**Figure 4.**
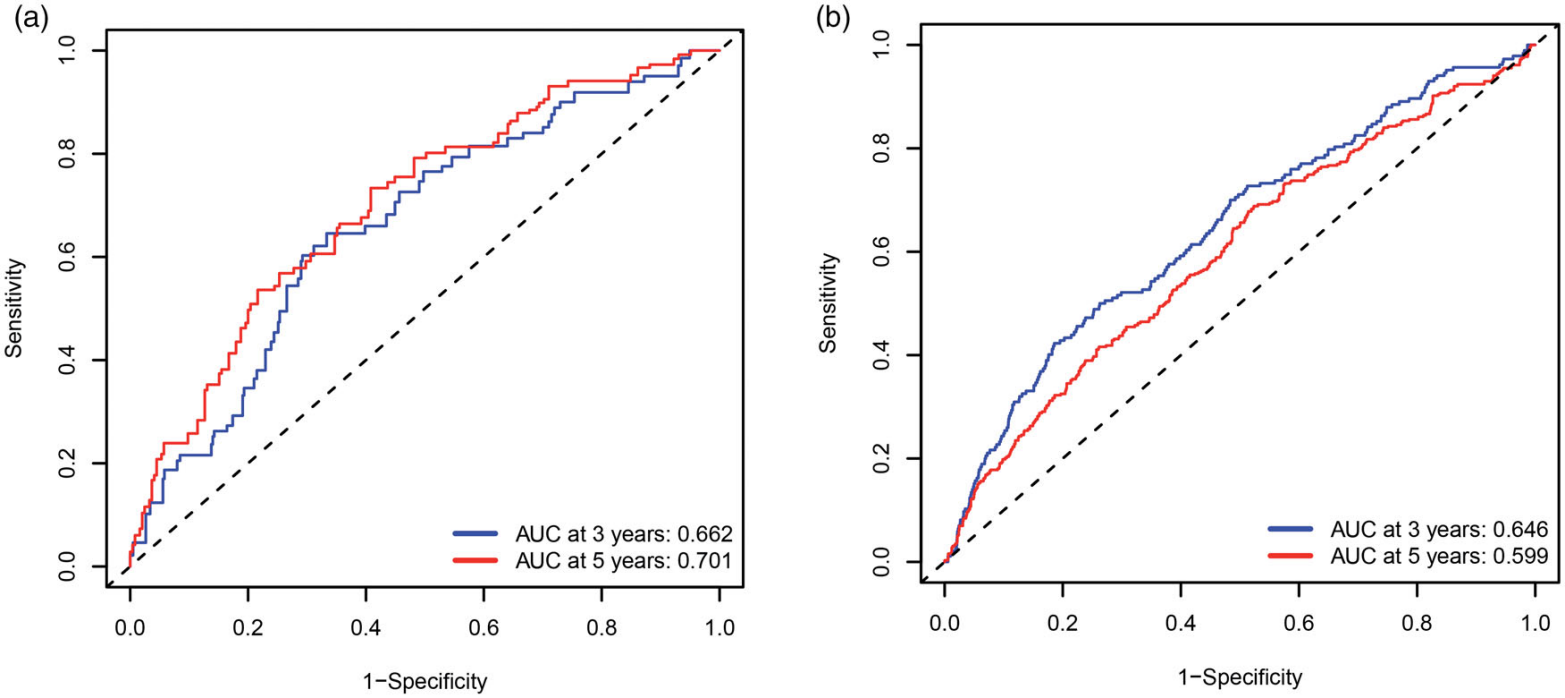
Receiver operating characteristic curves of the MMGS model to predict the 3- and 5-yearOS in the training set (a), validation set (b).

MMGS risk score was calculated for each individual patient from the TCGA cohort (Figure 3(a)). The heatmaps of model genes in the training set and the validation set are shown in Supplemental Figure S2(a, b), respectively. The cut-off of risk score (2.8425) generated by X-tile software was set to divide patients into high- and low-risk groups (Figure 3(b)). Patients in the high-risk group had a significantly shorter OS than those in the low-risk group (hazard ratio: 3.077 [95%confidence interval (CI): 1.912–4.954], *p* < .001) (Figure 3(c)). A time-dependent ROC analysis demonstrated that the AUC for 3-year and 5-year OS of this classifier were 0.662 and 0.701, respectively, indicating MMGS possesses good sensitivity and specificity in predicting the prognosis in training set (Figure 4(a)). The C-index of MMGS for OS prediction in training set was 0.666 (95%CI: 0.609–0.723).

### Validation of the prognostic value of MMGS

We first performed internal validation by bootstrap method (*B* = 1000), and the C-index of bootstrap validation of the prediction model was 0.667 (95% CI: 0.589–0.745), indicating good distinguishing capacity of this model. In an external independent cohort from the GSE96058 validation dataset, MMGS risk score was calculated for each individual patient (Figure 3(d)), and patients were classified into high- and low-risk subgroups based on the same risk score cut-off value previously mentioned (Figure 3(e)). The OS in the high-risk group was significantly shorter than that of the low-risk one (hazard ratio: 1.808 [95%CI: 1.458–2.243], *p* < .001) in the validation set (Figure 3(f)). AUC for 3-year and 5-year time-dependent ROC were 0.646 and 0.599, respectively (Figure 4(b)). The C-index of MMGS for predicting OS invalidation set was 0.618 (95%CI: 0.586–0.651).

### The correlation of MMGS risk score with classical clinical variables

**Figure 5.**
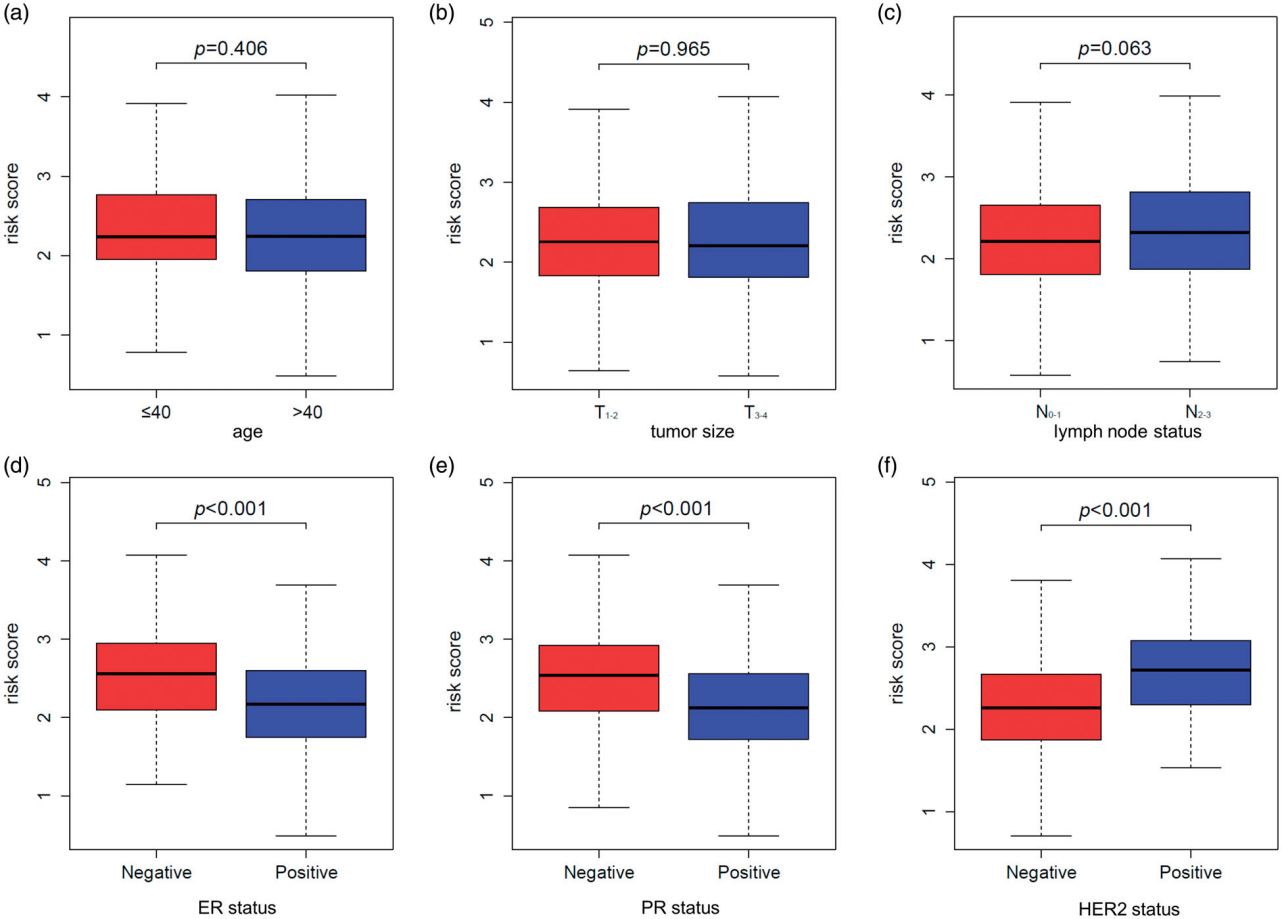
The relation between risk score and age (a), tumour size (b), lymph node status (c), ER status (d), PR status (e), HER2 status (f) of breast cancer patients in the TCGA database.

**Figure 6.**
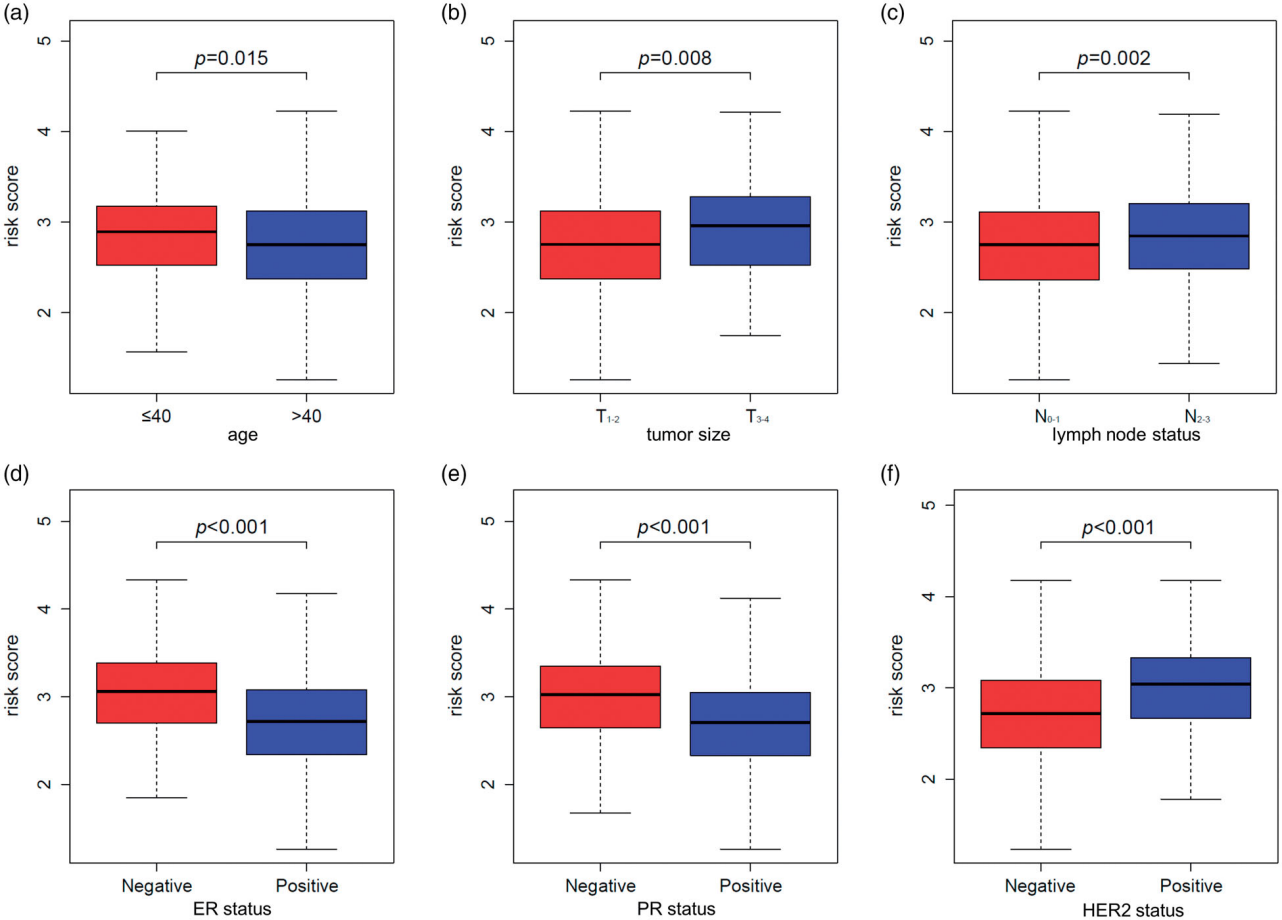
The relation between risk score and age (a), tumour size (b), lymph node status (c), ER status (d), PR status (e), HER2 status (f) of breast cancer patients in the GSE96058 validation dataset.

To investigate the correlation of MMGS risk score with clinicopathological factors, including age, tumour size, lymph node status, ER status, PR status, HER2 status, we calculated MMGS risk scores distribution in patients from TCGA database stratified by each clinical risk factors. We found the risk score was lower in the ER and PR positive groups while higher in HER2 positive group in training set (*p* < .001) (Figure 5). The same results were observed in the validation set (Figure 6). While the correlations of MMGS risk score with age, tumour size, lymph node status were inconsistent in training set and validation set (Figures 5 and 6). Then we explored the prognostic value of MMGS in different subgroups. In the training set, a significant poor survival probability was associated with high MMGS risk score in patients with different clinicopathological factors except age (≤40) and HER2 positive subgroups (Figure 7(a)). In the validation set, MMGS risk score effectively predict patients’ prognosis in hormone receptor positive and HER2 negative subgroups as well as all age and lymph node status subgroups (Figure 7(b)).

Figure 7.The correlation of MMGS risk score with classical clinical variables and the correlation between gene methylation and expression in MMGS. (a) The prognostic value of MMGS model in different subgroups in the TCGA training dataset. (b) The prognostic value of MMGS model in different subgroups in the GSE96058 validation dataset. (c) The correlation between DNA methylation and mRNA expression of CD74 and SERPINA1 in the risk score model.

**Figure:**
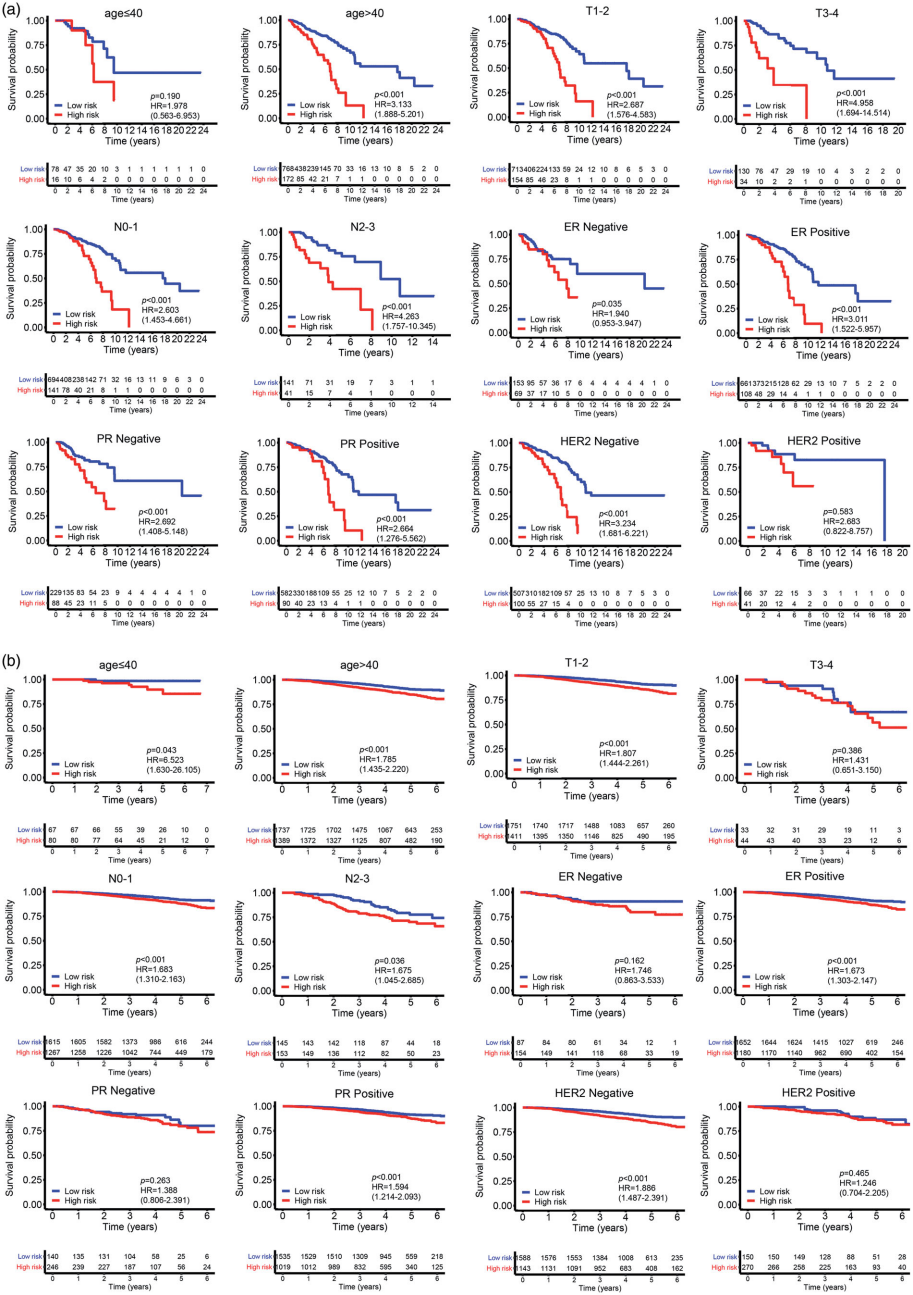

**Figure:**
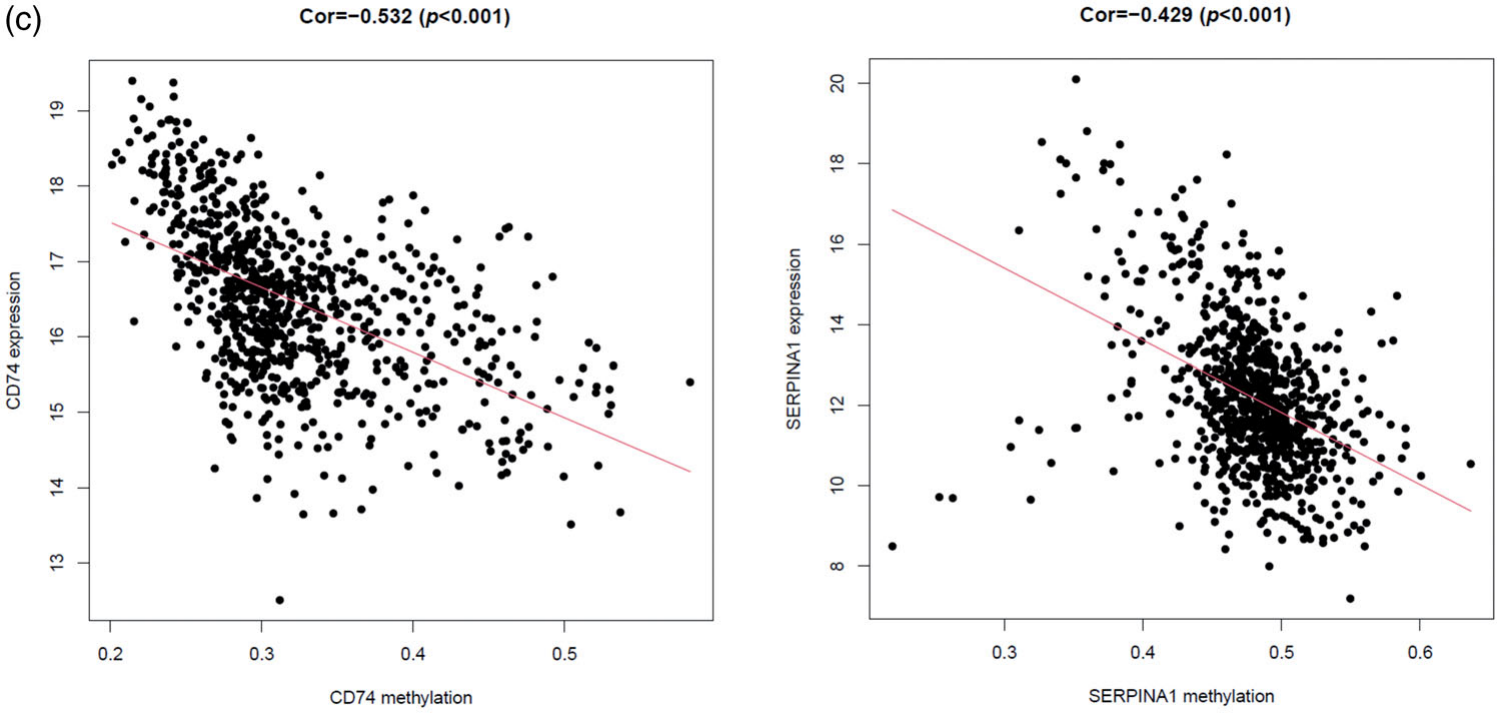

Next, we determine whether MMGS can be employed to independently predict the survival of breast cancer patients. MMGS and clinical characteristics, including age, tumour size, lymph node status, ER status, PR status, HER2 status, were included in univariate and multivariate analysis. MMGS remained an independent prognostic factor after adjusting for clinical characteristics in the multivariate analysis with the hazard ratio of 3.185 (95%CI: 1.983–5.115, *p* < .001) in training set (Table 1) and 1.362 (95%CI: 1.055–1.757, *p* = .018) in the validation cohort (Table 2).

**Table 1.** Univariate and multivariate analysis of breast cancer in the TCGA database.

Variables	Univariate analysis		Multivariate analysis	
	HR^a^ (95% CI)	*p* Value	HR^a^ (95% CI)	*p* Value
risk score (high *vs*. low)	3.172 (2.220–4.534)	<.001	3.185 (1.983–5.115)	**<.001**
age (y) (>40 *vs*. ≤40)	0.993 (0.589–1.672)	.978	1.024 (0.568–1.846)	.938
lymph node status (N_2-3_ *vs.* N_0-1_)	2.305 (1.547–3.433)	<.001	2.161 (1.285–3.635)	**.004**
tumour size (cm) (>5 *vs*. ≤5)	1.608 (1.093–2.364)	.016	1.432 (0.853–2.402)	.174
ER status (positive *vs.* negative)	0.711 (0.490–1.033)	.074	0.974 (0.484–1.963)	.942
PR status (positive *vs.* negative)	0.759 (0.535–1.077)	.123	0.890 (0.475–1.669)	.716
HER2 status (positive *vs.* negative)	1.024 (0.570–1.839)	.936	0.670 (0.339–1.327)	.251

^a^ Hazard ratio. *p* values less than 0.05 in multivariate analysis were highlighted in bold.

**Table 2.** Univariate and multivariate analysis of breast cancer in the GSE96058 dataset.

Variables	Univariate analysis		Multivariate analysis	
	HR^a^ (95% CI)	*p* Value	HR^a^ (95% CI)	*p* Value
risk score (high *vs*. low)	1.809 (1.456–2.247)	<.001	1.362 (1.055–1.757)	**.018**
age (>40 *vs*. ≤40)	1.868 (0.926–3.766)	.081	11.313 (1.584–80.792)	**.0156**
lymph node status (N_2-3_ *vs.* N_0-1_)	2.860 (2.191–3.735)	<.001	2.341 (1.701–3.221)	**<.001**
tumour size (>5cm *vs*. ≤5cm)	3.506 (2.332–5.270)	<.001	2.137 (1.279–3.571)	**.004**
ER (positive *vs.* negative)	0.524 (0.366–0.750)	<.001	0.735 (0.448–1.205)	.222
PR (positive *vs.* negative)	0.484 (0.361–0.648)	<.001	0.592 (0.392–0.895)	**.013**
HER2 (positive *vs.* negative)	1.197 (0.888–1.613)	.238	0.936 (0.643–1.365)	.733

^a^ Hazard ratio. *p* values less than 0.05 in multivariate analysis were highlighted in bold.

### The association between gene methylation and expression in MMGS

It is said that gene transcription could be regulated by DNA methylation level [22,23]. To lay the foundation for further research of these model genes, we obtained the gene methylation data from TCGA database and investigated the association between gene methylation and expression levels in the MMGS model. As shown in Figure 7(c), CD74 and SERPINA1 promoter hypermethylation is significantly correlated with lower gene expression (*p* < .001, cor= −0.532 for CD74 and *p* < .001, cor= −0.429 for SERPINA1), while STX11, ADAM9, NFKBIA, PGK1 promoter hypermethylation is not significantly correlated with gene expression (|r|<0.3, Supplemental Figure 3). CD24 promoter hypermethylation data were not obtained in the TCGA dataset, so the relationship remains unclear.

## Discussion

In this study, we performed bioinformatics analysis of breast cancer sc-RNA-seq profiles, and found out macrophage marker genes in the breast cancer tissue. The functions of macrophage marker genes are enriched in neutrophil activation, phagocytosis, macrophage activation, antigen processing and presentation, complement and coagulation cascades, etc. Then, we further constructed MMGS based on gene expression profiles and clinical information of breast cancer patients in the TCGA database and validated the MMGS with GSE96058 dataset. MMGS served as an independently prognostic factor of OS in breast cancer patients of both datasets. Besides, MMGS risk score is closely related to hormone receptors status. In hormone receptor positive and HER2 negative subgroup (HR+/HER2−), MMGS showed better distinguishing capability between high risk and low risk groups. It is suggested that macrophages play a pivotal role in breast cancer, especially in luminal subtypes.

In the past decades, multigene assays have been applied to instruct clinical practices. Currently, the two clinically available assays recommended by National Comprehensive Cancer Network (NCCN) guideline were Oncotype Dx and MammaPrint [34,35]. Oncotype DX relies on the genes that define the ER status, HER2 status, tumour proliferation, and tumour invasion. Compared with Oncotype DX, MammaPrint included genes associated with various pathways—such as adhesion and angiogenesis—as well as proliferation and HR-related genes. However, neither of them includes immune genes, especially specific immune cell marker genes. Even though immunotherapy becomes the promising management of prolonging overall survival of breast cancer patients, the biomarkers of immunotherapy efficacy and patients’ outcome are still uncertain. Here, we performed a detailed analysis of breast cancer scRNA-seq data, identified prognostic macrophage marker genes and further constructed a MMGS model, which is an independent factor of OS after adjusting classical clinical variables in both training and validation dataset. Besides, it is the first model constructed by using the specific immune cell marker genes expression profile and might reflect macrophages function in TME somehow. It is said that osteoclasts differentiate from monocytes/macrophages and exert great influences on osseous metastasis of breast cancer [36,37]. Based on the excellent distinguishing capability in HR+/HER2− subgroup, this model provides mechanism tips for osseous metastasis preference of HR+/HER2− subtype breast cancer. Further, we screened out survival-related macrophage marker genes and provides a basis for further study of the role of macrophages in the immune microenvironment and immunotherapy in the future.

Macrophage marker genes included in this model are: SERPINA1, CD74, STX11, ADAM9, CD24, NFKBIA, PGK1, indicating pivotal roles in macrophages’ biological behaviours. SERPINA1encodes a member of serine protease inhibitor superfamily, α1-antitrypsin (AAT), and is highly expressed in the liver and cultured hepatoma cells and, to a lesser extent, in macrophages. Independent of its primary function as a major inhibitor of proteases including neutrophil elastase (NE), SERPINA1is now recognized as immunomodulatory agent. It was reported to be the monocyte/macrophage featured gene involving in the host innate immune response against pathogen infections and facilitates macrophage polarization towards inflammatory phenotype [38]. Also, there were studies that showed AAT-treated macrophages exhibit a similar trend by polarizing towards the M2-like profile [39]. In the present study, we found low expression of SERPINA1 predicts patients’ poor outcome, indicating SERPINA1 functions anti-neoplastic roles in breast cancer. CD74 is the cognate receptor of macrophage migration inhibitory factor (MIF). MIF/CD74 is a well-established pro-tumorigenic signalling in several solid malignancies partially by participating in the alternate activation of tumour‐associated macrophages [40,41]. Syntaxin 11 (STX11) is found to be enriched in immune cells, including natural killer cells, cytotoxic T cells and monocytes/macrophages. In macrophagesSTX11 is located on endosomal membranes and lysosomes, functions in vesicular trafficking and secretion. Silencing of STX11 enhances the phagocytosis of apoptotic cells and antibody-dependent target cells, as well as the secretion of TNFα, suggesting an anti-tumoral effect of STX11 [42]. ADAM9 is known to be expressed by monocytes and activated macrophages. ADAM9 degrades several extracellular matrix (ECM) proteins indicating its pro-metastasis roles in tumour progression [43]. CD24 is a widely accepted cell surface marker for breast cancer stem cells. Its low or deficiency expression together with high CD44 level correlates with stem cell properties. Also, CD24 acts as a costimulatory molecule to promote adaptive immunity and sustain macrophage activity and survival during carcinogenesis [44]. NF-κB signalling is the central mechanism that maintains the alternative phenotype of TAMs and maintains the immunosuppressive phenotype of TAMs. NFKBIA (IκBα) that binds to and sequesters NF-κB in the cytoplasm is considered as a major brake on NF-κB signalling and TAM functions [45]. PGK1 is the first identified ATP-generating enzyme, which plays important role in regulating mitochondrial metabolism, thereby promoting tumorigenesis [46]. Nevertheless, functions of PGK1 in macrophage are largely unknown. Considering metabolic shifting between glycolysis and mitochondrial oxidative phosphorylation might determine macrophage polarization, PGK1 probably participants in macrophage activation [47]. Overall, the results of the present study may provide potential gene targets for prognostic evaluation to help improve the clinical outcomes of breast cancer.

Next, we analysed the correlation between gene expression level and DNA methylation of genes in MMGS. CD74 and SERPINA1 mRNA levels negatively correlated with DNA methylation, suggesting that DNA methylation may be one of the key regulators of these two genes’ transcription.

One of limitations of this study is that our study was based on retrospective cohorts. Besides, the interaction of macrophage marker genes with tumor-specific genes was not analysed in this study.

In conclusion, although there are still some limitations, our study provides a new understanding of immune cell marker genes in breast cancer prognosis and offers immunotherapy practices instructions for physicians.

## Supplementary Material

Supplemental Material
