# Supplementary material for: Bioinformatics reveal macrophages marker genes signature in breast cancer to predict prognosis
Source: Ann Med. 2021 Jun 30;53(1):1020–32. doi: 10.1080/07853890.2021.1914343 (PMC8253219; doi:10.1080/07853890.2021.1914343)
Supplement: Supplemental Material [file IANN_A_1914343_SM5338.zip › Supplemental files/Supplemental Table S1.docx]

**Table S1**. Characteristics of breast cancer cases in the TCGA and GSE96058 dataset

|  | TCGA (N=1034)  n (%) | GSE96058 (N=3273)  n (%) |
| --- | --- | --- |
| age (year) |  |  |
| ≤40 | 94 (9.1) | 147 (4.5) |
| >40 | 940 (90.9) | 3126 (95.5) |
| lymph node status |  |  |
| N_0-1_ | 835 (80.8) | 2882 (88.1) |
| N_2-3_ | 182 (17.6) | 298 (9.1) |
| NA | 17 (1.6) | 93 (2.8) |
| tumor size |  |  |
| T_1-2_ | 867 (57.2) | 3162 (96.6) |
| T_3-4_ | 164 (3.5) | 77 (2.4) |
| NA | 3 (0.3) | 34 (1.0) |
| ER status |  |  |
| positive | 769 (74.4) | 2832 (86.5) |
| negative | 222 (21.5) | 241 (7.4) |
| NA | 43 (4.2) | 200 (6.1) |
| PR status |  |  |
| positive | 672 (65.0) | 2554 (78.0) |
| negative | 317 (30.7) | 386 (11.8) |
| NA | 45 (4.4) | 333 (10.2) |
| HER2 status |  |  |
| positive | 107 (10.3) | 420 (12.8) |
| negative | 607 (58.7) | 2731 (83.4) |
| NA | 320 (30.9) | 122 (3.7) |

NA: not available
