# Supplementary figures and images for: Bioinformatics reveal macrophages marker genes signature in breast cancer to predict prognosis
Source: Ann Med. 2021 Jun 30;53(1):1020–32. doi: 10.1080/07853890.2021.1914343 (PMC8253219; doi:10.1080/07853890.2021.1914343)

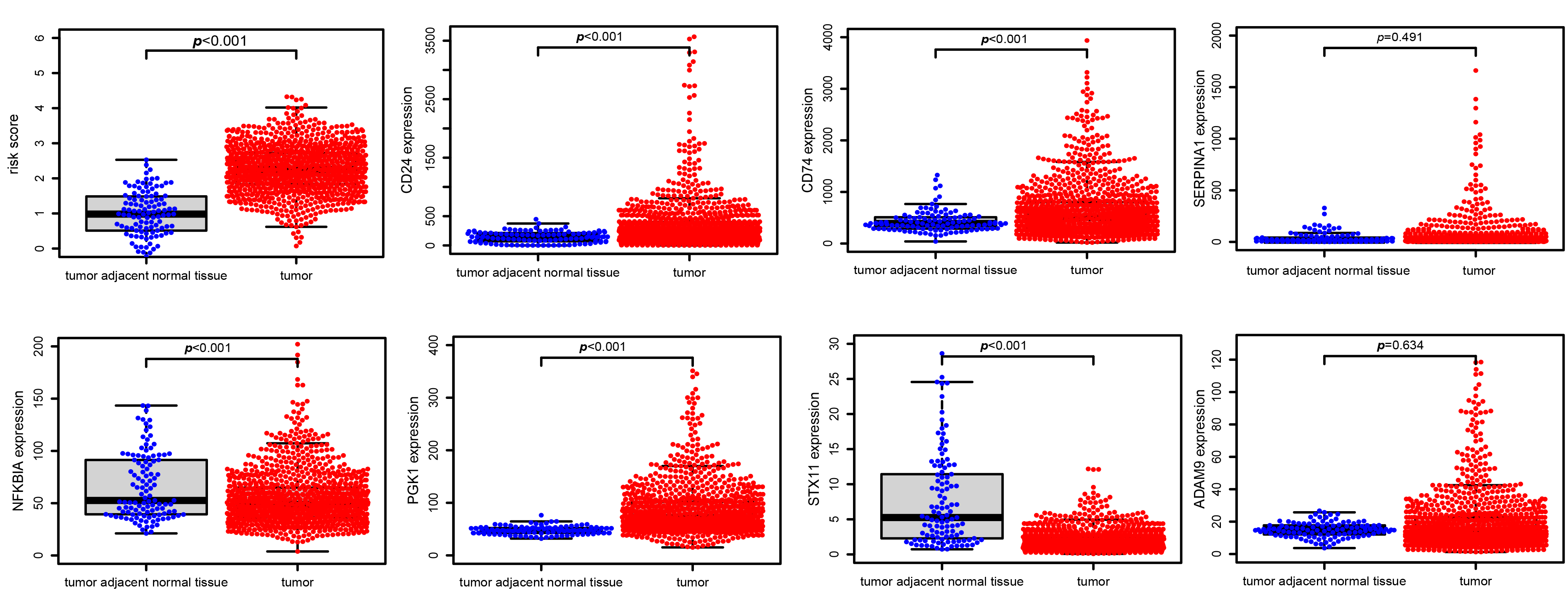

Supplement: Supplemental Material [file IANN_A_1914343_SM5338.zip › Supplemental files/supplemental Figure S1.tif]

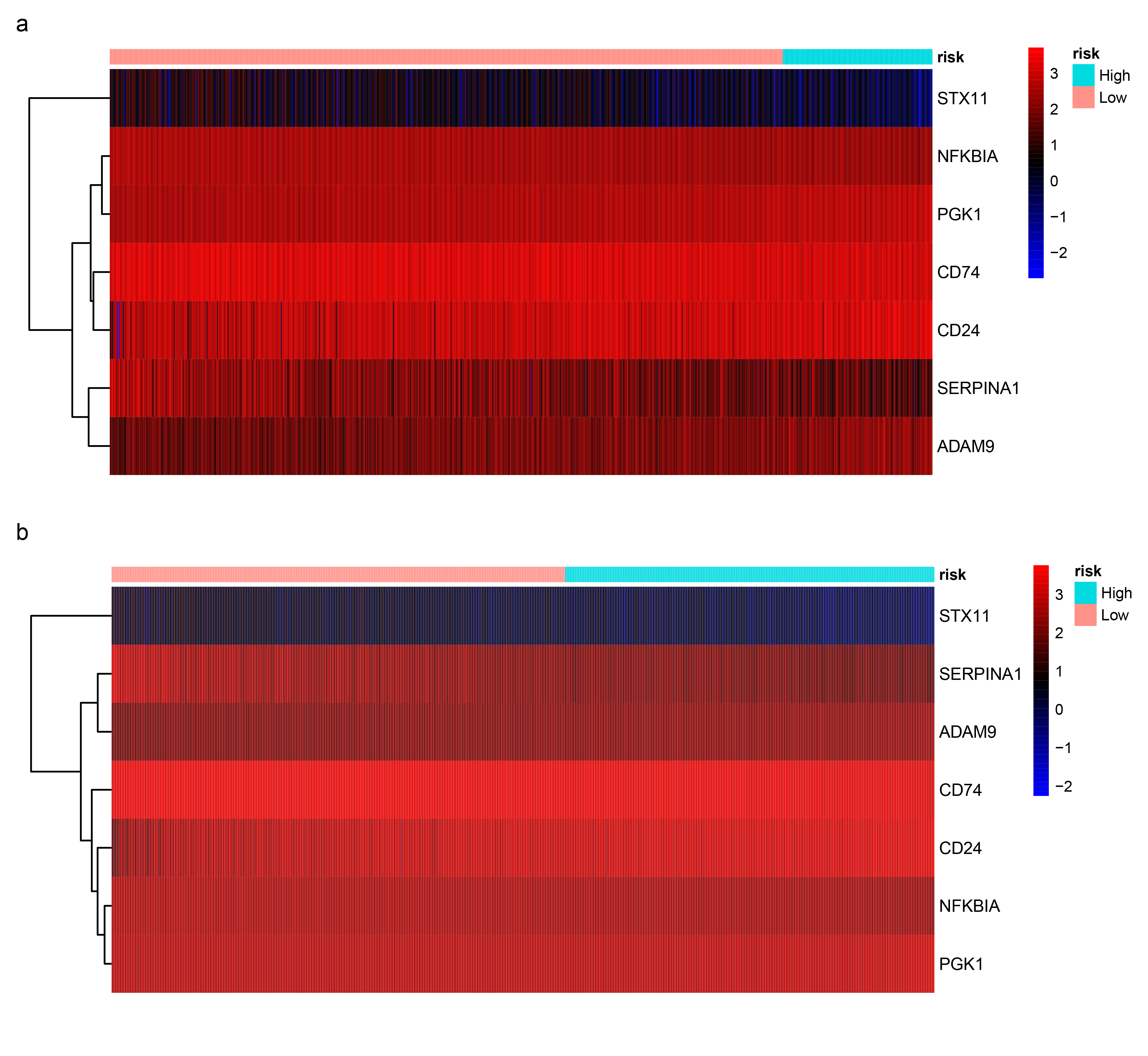

Supplement: Supplemental Material [file IANN_A_1914343_SM5338.zip › Supplemental files/supplemental Figure S2.tif]

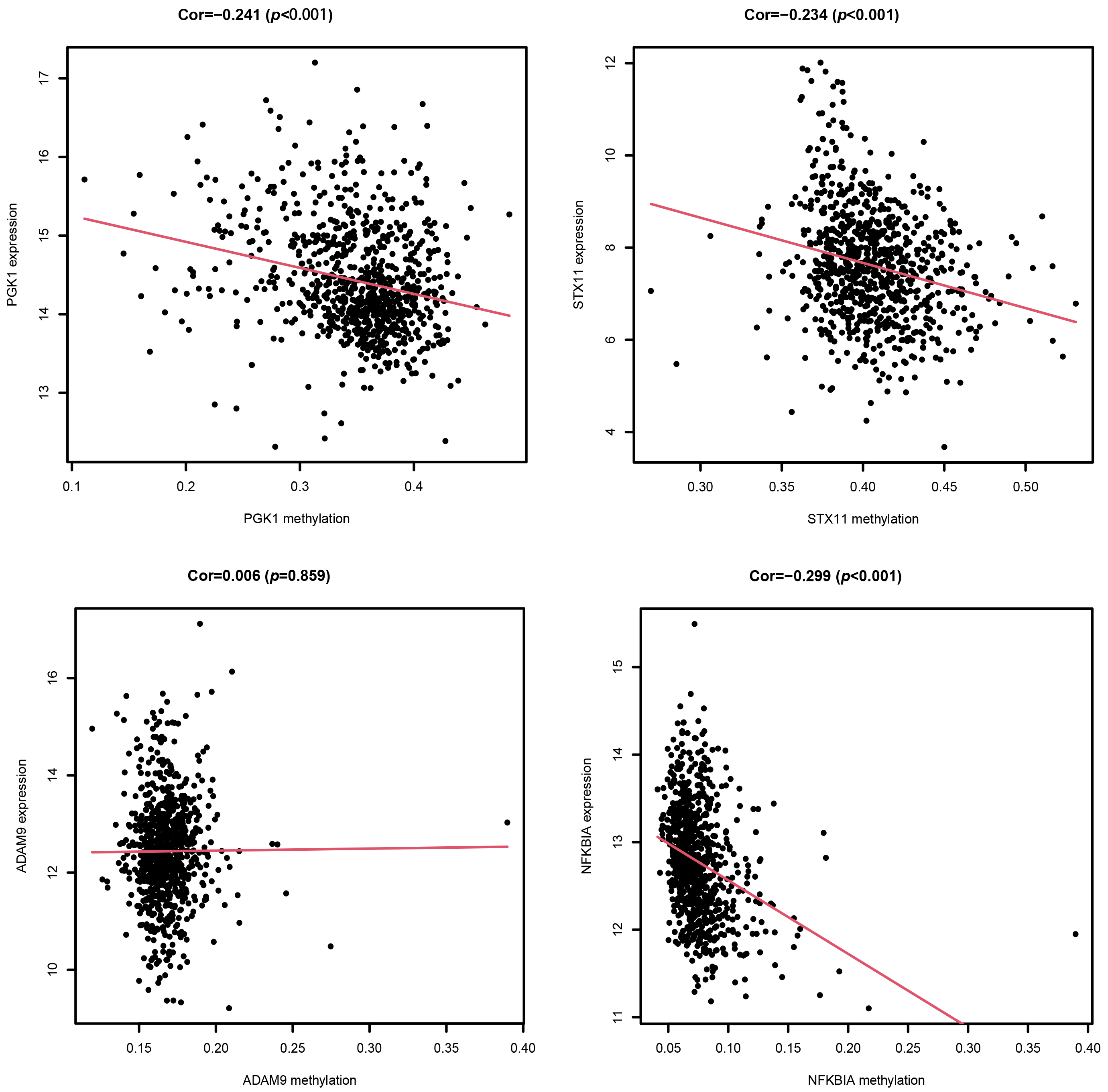

Supplement: Supplemental Material [file IANN_A_1914343_SM5338.zip › Supplemental files/supplemental Figure S3.tif]
